# Scaling Up of an Innovative Intervention to Reduce Risk of Dengue, Chikungunya, and Zika Transmission in Uruguay in the Framework of an Intersectoral Approach with and without Community Participation

**DOI:** 10.4269/ajtmh.17-0061

**Published:** 2017-08-07

**Authors:** César Basso, Elsa García da Rosa, Rosario Lairihoy, Ruben M. Caffera, Ingrid Roche, Cristina González, Ricardo da Rosa, Alexis Gularte, Eduardo Alfonso-Sierra, Max Petzold, Axel Kroeger, Johannes Sommerfeld

**Affiliations:** 1 Unidad de Entomología, Departamento de Protección Vegetal, Facultad de Agronomía, Universidad de la República, Montevideo, Uruguay;; 2 Departamento de Parasitología Veterinaria, Facultad de Veterinaria, Universidad de la República, Salto, Uruguay;; 3 Departamento de Sistemas Ambientales, Facultad de Agronomía, Universidad de la República, Montevideo, Uruguay;; 4 Instituto de Teoría de la Arquitectura y Urbanismo, Facultad de Arquitectura, Diseño y Urbanismo, Universidad de la República, Montevideo, Uruguay;; 5 Dirección Departamental de Salud de Salto, Ministerio de Salud Pública, Salto, Uruguay;; 6 Centre for Medicine and Society/Anthropology, Freiburg University, Freiburg, Germany;; 7 Centre of Applied Biostatistics, The Sahlgrenska Academy, University of Gothenburg, Gothenburg, Sweden;; 8 Liverpool School of Tropical Medicine, Liverpool, United Kingdom;; 9 Special Programme for Research and Training in Tropical Diseases (TDR), World Health Organization (WHO), Geneva, Switzerland

## Abstract

To contribute to the prevention of dengue, chikungunya, and Zika, a process of scaling up an innovative intervention to reduce *Aedes aegypti* habitats, was carried out in the city of Salto (Uruguay) based on a transdisciplinary analysis of the eco-bio-social determinants. The intervention in one-third of the city included the distributions of plastic bags for all households to collect all discarded water containers that were recollected by the Ministry of Health and the Municipality vector control services. The results were evaluated in 20 randomly assigned clusters of 100 households each, in the intervention and control arm. The intervention resulted in a significantly larger decrease in the number of pupae per person index (as a proxy for adult vector abundance) than the corresponding decrease in the control areas (both areas decreased by winter effects). The reduction of intervention costs (“incremental costs”) in relation to routine vector control activities was 46%. Community participation increased the collaboration with the intervention program considerably (from 48% of bags handed back out of the total of bags delivered to 59% of bags handed back). Although the costs increased by 26% compared with intervention without community participation, the acceptability of actions by residents increased from 66% to 78%.

## INTRODUCTION

*Aedes aegypti* (L.) (Diptera: Culicidae) is the major urban vector of dengue viruses (DENV) worldwide. Over the last 25 years, there has been a global increase in both the distribution of *Ae. aegypti* and epidemic DENVs activity.^1^ Over half of the world’s population inhabit areas at risk of dengue infection.^2,3^ Currently, the World Health Organization (WHO) reports its presence in more than 125 countries^4^ and recent modeling suggests that as many as 390 million infections occur annually.^5^ The Region of the Americas is not an exception; between 2010 and 2016, more than 1.7 million cases of dengue were notified annually, including 24,500 severe cases and 1,000 deaths.^6^ In recent years, Zika virus (ZIKV) and chikungunya virus (CHIKV), two emerging mosquito-borne flaviviruses also transmitted by *Ae. aegypti*, showed a dramatic increase in the Americas. Brazil is the most affected country, with 109,596 confirmed cumulative cases of autochthonous ZIKV infection reported in 2015–2016,^7^ but others countries are affected or with a high risk of being affected by these diseases.^7–10^

*Aedes aegypti* is closely associated with human habitation. Females preferentially lay eggs in manmade containers including water tanks, flower vases, pot plant bases, discarded tires, buckets, or other containers typically found around or inside the home.^11^ Eggs are laid on the walls of containers near the water surface and, once embryonated, can withstand desiccation for up to 1 year.^12^ Particular features that have been observed as associated with *Ae. aegypti*’s presence include urbanization, socioeconomic factors, building design and construction features, the quality of water supply and management, and the quality of other public-health infrastructure services.^13,14^

DENV, ZIKV, and CHIKV prevention and mitigation are closely associated with human actions aimed at reducing the presence and abundance of *Ae. aegypti*. Multifactorial determinants related to vector’s ability of transmitting diseases, demand an ecosystemic approach that considers eco-bio-social factors affecting human health, called Ecohealth by Lebel.^15^ Vector control tools, regardless of their technological basis, must be feasible and practical to apply in real-life situations. Community engagement and intersectoral partnerships are particularly important elements of integrated public health strategies against this vector.^16^

In Uruguay, a country located on the southern boundary of *Ae. aegypti*’s distribution in South America,^17^ *Aedes aegypti* was detected in 1997 after almost 40 years of absence.^18^ Since then its dispersion has steadily increased and now occupies much of the national territory.^19^ Uruguay is surrounded by dengue endemic areas (Argentina and Brazil)^9,20^ and in February 2016, the first 26 autochthonous cases appeared.^6,19^

Because of its geographic location, Uruguay has long periods during which temperatures fall below oviposition and activity thresholds of *Ae. aegypti*.^11,21,22^ The vector population grows when temperature rises, resulting in a particular population dynamic only occurring during the hot season.^23^

Dengue entomological surveillance is using since the 1960s larval indices for determining presence or absence of the vector (Stegomyia indices: the Container Index-CI, the House Index-HI, and the Breteau Index-BI). However, these indices do not reflect vector abundance and do not identify those water containers, which are most productive for the adult stage of the vector. Chadee and Focks^24,25^ merged the concept of key premises and productive containers with the pupal indices,^26^ which reflect the adult productivity of different container types. The concept of container productivity and the risk of disease transmission based on the number of people living in each house (number of pupae per person index-PPI) may serve as an improved indicator in ecological settings where DENV is an important public-health problem. The PPI indicator can then be used as an outcome measure for targeted approaches to vector control and DENV suppression programs.^27–32^

The efficacy of an innovative *Ae. aegypti* intervention package was tested from 2011 to 2013 through cluster-randomized controlled trials in the city of Salto in Uruguay following a Research and Training in Tropical Diseases International Development Research Centre research initiative that included other four cities in the Americas. A collaborative partnership between the Ministry of Health, Municipality of Salto, and a project team from the University of the Republic was the key to this initiative.^33^ In the first phase of the study, the innovative intervention saved costs compared with the routine activities reducing the cost per house attended by nearly 21% and reducing dengue vector densities (although not to statistically significant levels). These promising results justified a new step for a considerable scaling up of the experiences. Scaling up refers to planned efforts to increase the impact of successfully tested health interventions to benefit more people and promote public policy from this example. The effectiveness of the approach and peoples’ acceptance were major outcome indicators in the here presented study.

## MATERIAL AND METHODS

### Study area, study design, and sampling.

The location of this study includes the whole urban area of the city of Salto located in northwestern Uruguay (31°23ʺS, 57°58ʺW). The city has 123,000 inhabitants. The climate is such that vectors survive long enough to complete the viral extrinsic incubation period during about 5 months of the year. Local arbovirus transmission is biologically possible only during this period.^23,34^ Dengue herd immunity in the urban population of Salto can be considered to be close to zero as there has been no reported virus transmission in recent years.

**Figure 1.**
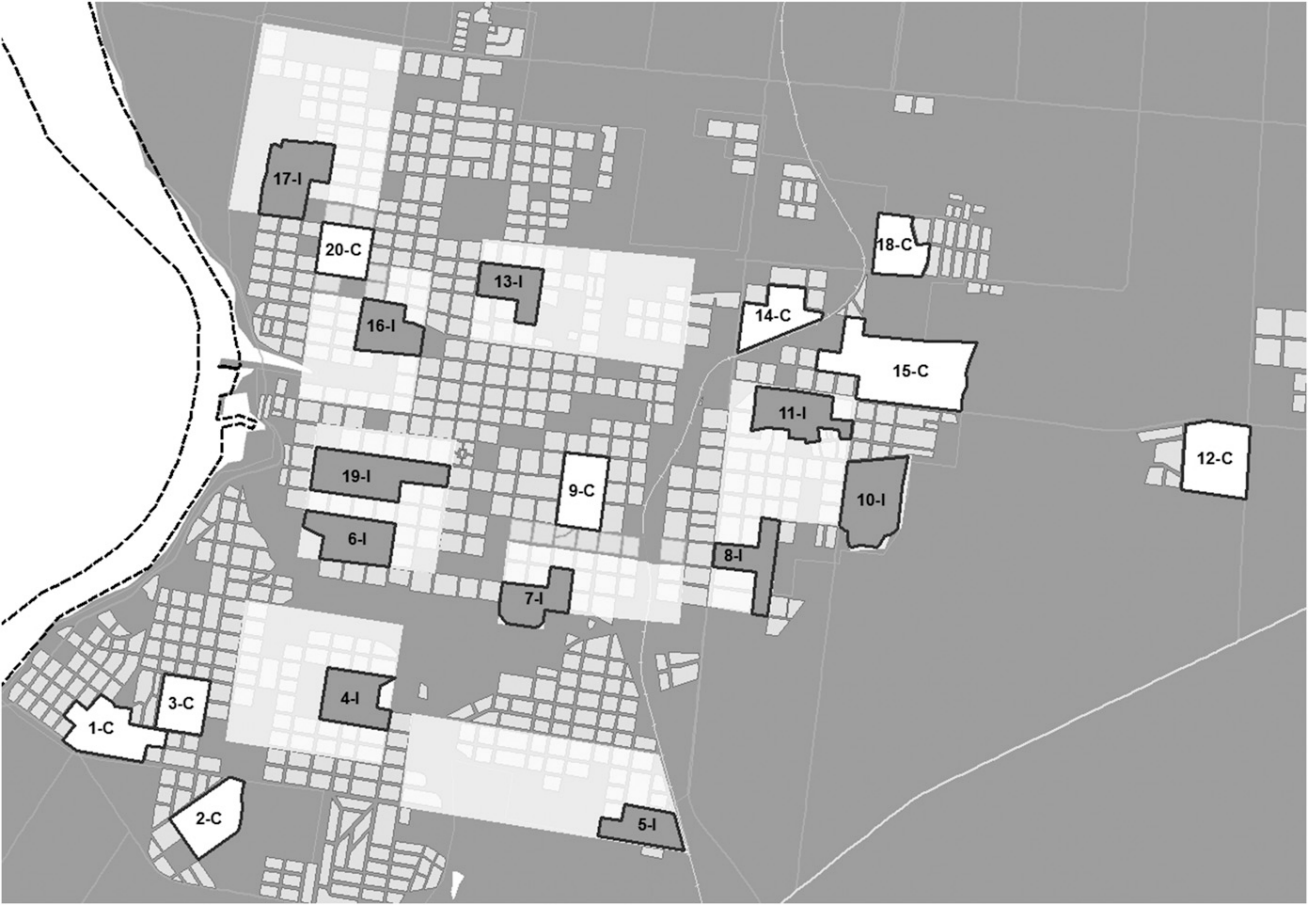
Eight areas of intervention in the city of Salto where the scaling up process was carried out. Eleven clusters (I) were included in those eight areas. Outside those areas, nine clusters were kept as controls (C).

Various clusters or cells of Salto were delimited by overlaying a sampling grid with 200 cells on a geo-referenced digital map of this city, using ArcGIS 9.3 (Redlands, CA). A cluster was defined as a geographical area that includes at least 100 private households. The clusters were numbered and 20 clusters were randomly selected using simple random numbers. Among them, 11 intervention and nine control clusters were randomly selected. Later, eight areas that included the intervention clusters were defined including in total 10,000 residences (about one-third of the city of Salto) (Figure 1). The dengue intervention activities took place in those eight areas, whereas the entomological and knowledge and satisfaction surveys to evaluate the effect of the intervention were done only in the intervention clusters. The other nine clusters were kept as controls. Within the control clusters, the Ministry of Health went on with routine activities to keep the vector under control, which involves entering the premises to collect and remove the water containers. Care was taken to ensure that the control clusters were at least 200 m (which is beyond the usual flight range of *Aedes* mosquitoes) from the nearest intervention areas to avoid any spill-over effects (Figure 1).

The clusters belonging to intervention and control areas shared, on the whole, similar characteristics in terms of geography and ecology (flooding zones and areas not subject to flooding, abundant, or scarce vegetation); housing types; economic, cultural, and social aspects (lower, middle, and high socioeconomic levels); as well as the ecological situation and the socioeconomic characteristics of the population.

The study was conducted from April to November 2015 and involved researchers from the University of the Republic of Uruguay, health professionals from the Ministry of Health and of the Municipality of Salto and international experts.

### Vector control interventions and evaluation of their acceptance by the community.

Ecosystem management measures consisted of promoting and organizing a campaign together with public-health institutions for the physical removal of containers in all homes located in the eight intervention areas. In one of the areas (Area 7), an enhanced social mobilization process based on active community participation was implemented. Households in the intervention areas were surveyed within 5 months. From June to October 2015, employees assigned by the Ministry of Health and paid by the Ministry of Social Development (MIDES) and the Municipality of Salto visited households informed about the purpose of the intervention and handed over a plastic bag for collecting unused small containers (garbage collection). The household members had 1 or 2 days for completing the collection. The municipal workers were supposed to transport the bags with discarded containers to a municipal collection point where they were recycled. However, as a result of a difficult political situation, the Municipality did not collaborate at this point in time. Therefore the transport of the bags with discarded containers was contracted out with a private company.

During the implementation of the intervention messages were transmitted to the population through the radio, television, and written press. In these messages, it was detailed what was the zone of the city where the activity took place and the characteristics of the activity.

For further analysis of the intervention package, a household survey was conducted in all intervention clusters to find out if the households had taken part in the activity. In case of a positive response, information was gathered about the opinion of the residents, if they knew about the activity beforehand, how they had learned about it, and if they were willing to participate again in the future.

Also in the control clusters a household survey was conducted to know whether the interviewees had heard that an activity consisting in delivery of bags to remove containers form households had been carried out in other areas of Salto. If the answer was positive, it was asked how the person found out and also if he/she was willing to participate in a similar activity in the future.

### Community and stakeholder engagement.

Seeking community participation in the intervention planned, in one of the eight areas (Area 7), many activities with social groups, schools, and community organizations were organized during 4 weeks before the delivery of bags for the containers collection. In this activity, it was used a procedure similar to that done in other areas. Different ways to share information and promote mobilization of the population (according to principles formulated by Draper and others^35^) were developed (meetings with teachers, parents, students, representatives of different community organizations, physicians...).

In the week in which the containers were collected, in Area 7, a car with a loudspeaker drove repeatedly broadcasting a message about the activity. Through that publicity, it was desired to inform the neighbors that the activity was occurring at that time.

Many of the organizations and institutions which were involved in the project are members of the SOCAT COVIFOE-COVISUNCA (Consultancy and Orientation Services of the Ministry for Social Development MIDES), a discussion and an executive group which meets monthly and provided the opportunity for introducing the project.

During the week preceding the intervention activities in this area (similar to those performed in the other areas), a household survey was executed, aimed at evaluating the information level of the neighbors about the activity. That information level should reflect the activity of the involved social organizations. Students and employees of the Social Ministry MIDES visited eight homes in each block (two in each street section) out of a total of 28 house blocks—half of the total area under research. Residents were asked if they knew about the activity that would take place in their neighborhood.

### Entomological surveys.

Entomological surveys identifying and quantifying larvae and pupae of *Ae. aegypti* were carried out in the study clusters including intervention and control areas. The surveys determined presence or absence of larvae, and pupae count per container in all water-filled containers present in peridomestic environment of households. Surveys were carried out in two stages: from April 19 to May 24, 2015 (baseline values before intervention activities; elevated vector densities due to favorable climatic conditions in late summer) and from November 2 to 30, 2015 (follow-up values after intervention activities; spring in the Southern Hemisphere, low vector density due to low temperatures in the preceding winter). Field work was conducted by 14 people trained and supervised by the project team members and by officials of the Departmental Health Section of Salto (DHSS) of the Ministry of Health.

The containers were counted and classified according to their size and use (in use = routinely used; not in use = abandoned or stacked^33,34^; Table 1). Only wet containers were recorded.

**Table 1:** Number of containers and mean number of pupae by type of containers collected in baseline (April-May 2015, autumn; elevated vector densities due to higher temperatures) and follow up (November 2015, spring; low vector density due to low temperatures)

Container type	Intervention clusters				Control clusters			
	Baseline		Follow-up		Baseline		Follow-up	
	Container *n* (%)	Mean pupae/container	Container *n* (%)	Mean pupae/container	Container *n* (%)	Mean pupae/container	Container *n* (%)	Mean pupae/container
Tanks*	39 (3.2)	0.46	51 (3.4)	0.08	39 (3.9)	0.21	45 (3.8)	0.39
Large standing cement water tanks*	6 (0.5)	0	19 (1.3)	0	18 (1.8)	0.11	26 (2.2)	0
Paint can-sized water containers*	7 (0.6)	0	9 (0.6)	0	9 (0.9)	0	1 (0.1)	0
Buckets*	82 (6.8)	0.05	95 (6.4)	0.09	63 (6.3)	0.06	62 (5.2)	0.08
Others*	960 (79.8)	0.01	1182 (79.6)	0	744 (74.5)	0.05	997 (84.3)	0.00
Paint can-sized water containers†	11 (0.9)	13.50	15 (1.0)	0.53	5 (0.5)	0.40	6 (0.5)	0
Flower vases†	1 (0.1)	0	1 (0.1)	5.00	0 (0.0)	0	2 (0.2)	0
Tires†	20 (1.7)	1.24	8 (0.5)	0.38	10 (1.0)	0.75	4 (0.6)	0
Bottles†	7 (0.7)	0	49 (3.3)	0	46 (4.6)	0	8 (0.7)	0
Other small miscellaneous containers†	36 (3.0)	2.06	33 (2.2)	0	31(3.1)	1.00	9 (0.8)	0.17
Tanks†	6 (0.5)	1.80	4 (0.3)	0	11 (1.2)	0.40	4 (0.3)	0
Other large miscellaneous containers†	28 (2.3)	0.71	19 (1.3)	0	23 (2.3)	0.39	16 (1.4)	0
Total	1,203 (100)	0.26	1,485 (100)	0.02	999	0.09	1,183	0.02

Number of house: Intervention clusters: 809, Control clusters: 585 (in both surveys).

* In use.

† Not in use.

All larvae and pupae found were stored in small vials with alcohol (identifying the container they came from) and transported to the laboratory of the DHSS, where they were identified using the Darsie key^36^ and counted. The primary outcome measure for determining the impact on vector population was the reduction of the PPI in intervention versus control clusters. Secondary outcome indicators (which however do not measure vector densities^24^) were the larval indices (CI, HI, and BI).

### Spatial representation.

To represent the pattern of spatial distribution of events (PPI) based on the corresponding coordinates, we used data interpolation and data smoothing using the Gaussian kernel.^37^ This method allows estimating the probability of the occurrence of an event in each cell of a regular grid, with each cell of this grid being the weighted average of all values for that site. These values are assigned using a probability distribution function—in this case Gaussian. The degree of smoothing is controlled by choosing a bandwidth which indicates the area to be considered in the calculation. This area should be related to the geographic scale of the hypothesis of interest or to prior knowledge about the problem under study.^38^ In agreement with Souza-Santos and Carvalho,^37^ this analysis used a bandwidth of 300 m based on dispersion of the female *Ae. aegypti* when they are not able to find suitable containers for females oviposit.^39^

### Cost analysis.

The costs of the interventions were analyzed from the perspective of the agencies in charge of vector control (Ministry of Health and Municipality of Salto). We used a microcosting approach^40^ and identified resources consumed for each activity. Data collection tools were developed to measure resource consumption in physical units and value each resource item at their unit costs. We classified the cost item following categories proposed in the literature.^41,42^ We collected information on personnel in terms of working hours to perform vector control activities and salaries, transport costs by measuring kilometers traveled and using average fuel consumption and market prices for fuel. We also measured quantities of consumables used and their unit costs and the expenses incurred in meetings. We did not include overhead (joint) costs in the analysis. Comparable information was obtained from the Ministry of Health and Municipality of Salto (routine activity).

Personnel from public agencies were primarily responsible for delivering the intervention. However, the research team also conducted key activities. In these cases, we included the time devoted to the intervention by research team members but for the cost analysis we took the salaries from personnel of the public sector. This was to avoid over estimation of costs due to relatively higher salaries of researchers.

As explained previously, the transport of the bags with discarded containers was contracted out with the private sector, which increased the costs of delivering the intervention. This increased cost, however, is not expected to occur in the routine implementation of the interventions. Therefore, in the costs estimates we used the number of kilometers traveled during the intervention and valued it at the unit cost for the public sector (recurrent costs) instead of the costs actually paid to the private enterprise. We also included the capital costs of the vehicles and the personnel required to operate them and actually transport the bags (not included in a scenario considering the costs of contracting out transport, because in the unit cost paid personnel were already included as well as capital costs).

For capital costs we included only vehicles, because for items such as uniforms we assumed only 1-year useful lifetime, therefore, they become a recurrent cost. We obtained equivalent annual costs by an annuitization procedure using 3% discount rate. For old vehicles and equipment, we used the replacement cost of the equipment, full useful life and 20% resale value and we allocated to the intervention or routine proportional to the fraction of time used on the intervention. Costs were estimated in local currency and converted to United States dollar (USD) using the average exchange rate during 2015 (27.27 Uruguayan Peso/USD).

Costs of the intervention process (with and without community participation) and routine activities executed by the Ministry of Health and Municipality of Salto were compared with costs of intervention activities executed previously in the same city only at clusters level^33^ to check if there were differences due to the scale.

### Statistical analysis.

Differences between intervention and control areas were tested using *t* test: confidence intervals were calculated based on the normal distribution. The difference in change from baseline to follow-up between intervention and control areas was estimated using an interaction effect in a linear regression. The interaction was 1 for observation in the intervention group at follow-up and 0 otherwise. The interaction effect is then (Follow-up value–Baseline value) for the intervention – (Follow-up value–Baseline value) for the control. A negative values means that the decrease in the intervention area is larger than in the control area.

### Ethical approval.

Informed consent was sought from all interviewees and anonymity was assured with respect to recorded and reproduced interview data. Ethical approval was obtained from WHO Ethical Review Committee and the Institutional Review Board of the Universidad de la República.

## RESULTS

### Container classification, productivity, and *Ae. aegypti* indices.

Pupal surveys carried out in the premises of study clusters but also in other areas of the city allowed identifying the most productive container types (survey 1 during the warm season = 2,202 water holding containers; survey 2 during spring = 2,668 water holding containers). Unused containers had the highest productivity of *Ae. aegypti* pupae (as a proxy for adult abundance): 0.83 pupae/unused container versus 0.02 pupae in the containers in use. The water containers not in use included flower vases, tires, paint can–sized containers, bottles, tanks, and miscellaneous small containers. The containers not in use represented 8.5% of the total number of containers but they produced 76% of all pupae collected. Paint-can sized water containers (capacity: 5–10 L, not in use) were the most productive containers not in use (4.65 pupae/container) and although they represented only 0.8% of the total number of containers, they produced 38% of all pupae collected. Meanwhile, “others small miscellaneous containers not in use” with capacity < 5 l (2.2% of total number of containers) produced 21% of pupae. Tanks not in use showed twice the productivity compared with tanks in use (0.52 pupae/tank versus 0.27 pupae/tank, respectively). The containers usually used at homes (large standing cement wash tanks, paint can–sized water containers, buckets) had very low pupal productivity (they produced only 6% of the total number of pupae; Table 1).

### Conducting the intervention.

A total of 9,111 houses were visited within the defined areas (91% of the target houses). Bags for the collection of discarded containers were delivered to 58% of those houses (5,319). In the remaining houses, residents were not available at the time of the visit and repeated visits. Of the bags delivered, 86% (which correspond to 4,574 houses = 86% of 5,319) were collected within 2–7 days, of which 58% were filled with discarded containers (2,631/4,574). The remaining 42% (1,943/4,574) were returned empty as the occupants stated that they had no containers in their homes. This indicates that 29% of houses visited had containers which were removed (2,631/9,111) and collected by a truck. In many homes with discarded containers two or three bags were filled and collected, in total 1,248 additional bags were collected thus increasing the number of filled bags by 47%.

Sometimes the collection truck did not arrive on time and in one area local dwellers demanded the truck to collect also other waste which delayed the removal.

### Community involvement.

Twelve activities, involving 238 persons—besides the project team members—took place to share information and promote mobilization of the residents in area 7. A household survey about peoples’ willingness to collaborate with the project was conducted before the intervention but after promotional activities in the intervention clusters. It showed that 37% of the households visited (79/215) had received information about the planned activities.

Teachers from a Center of Support for Childhood and Families not included in the intervention area showed interest in learning about the methodology used by the project. They were interested in duplicating it on their own at their Center, with support from students and parents.

### Entomological impact of the intervention.

The analysis of entomological indices at baseline and at follow-up 1 month (November 2015) after the intervention is shown in Table 2. As already mentioned, the vector population in Uruguay shows seasonal variations according to fluctuations in temperature leading to marked reductions in winter and to an increase from spring onward with the highest values in autumn. When comparing the variation from autumn 2015 (baseline) to spring 2015 (follow-up) the vector densities in intervention clusters on average decreased more than those in the control clusters. As an example, the average PPI (as the best proxy measure for adult vectors) decreased in the intervention clusters 11 times and in the control clusters only four times (*P* < 0.05). The CI, HI, and BI decreased in the intervention clusters more than those in the control clusters, although the difference was statistically not significant probably due to the small sample size of clusters.

**Table 2:** Analysis of the CI, HI, BI, and number of PPI values obtained in autumn (April 2015; elevated vector densities due to higher temperatures) to spring (November 2015; low vector density due to low temperatures) in intervention and control clusters

	Intervention		Control		Difference in decrease (*P* value*)
	Baseline	Follow up	Baseline	Follow up	
CI	5.15	0.97	7.54	1.83	1.52 (0.56) NS
HI	4.83	1.78	8.45	2.71	2.68 (0.30) NS
BI	7.09	1.89	10.72	3.07	2.45 (0.50) NS
PPI	0.110	0.010	0.050	0.013	−0.06 (0.042) S

A negative values means that the decrease in the intervention area was larger than in the control area.

BI = Breteau index; CI = Container index; HI = House index; NS: not significant; PPI = pupae per person index; S: significant.

* *P* < 0.05.

**Figure 2.**
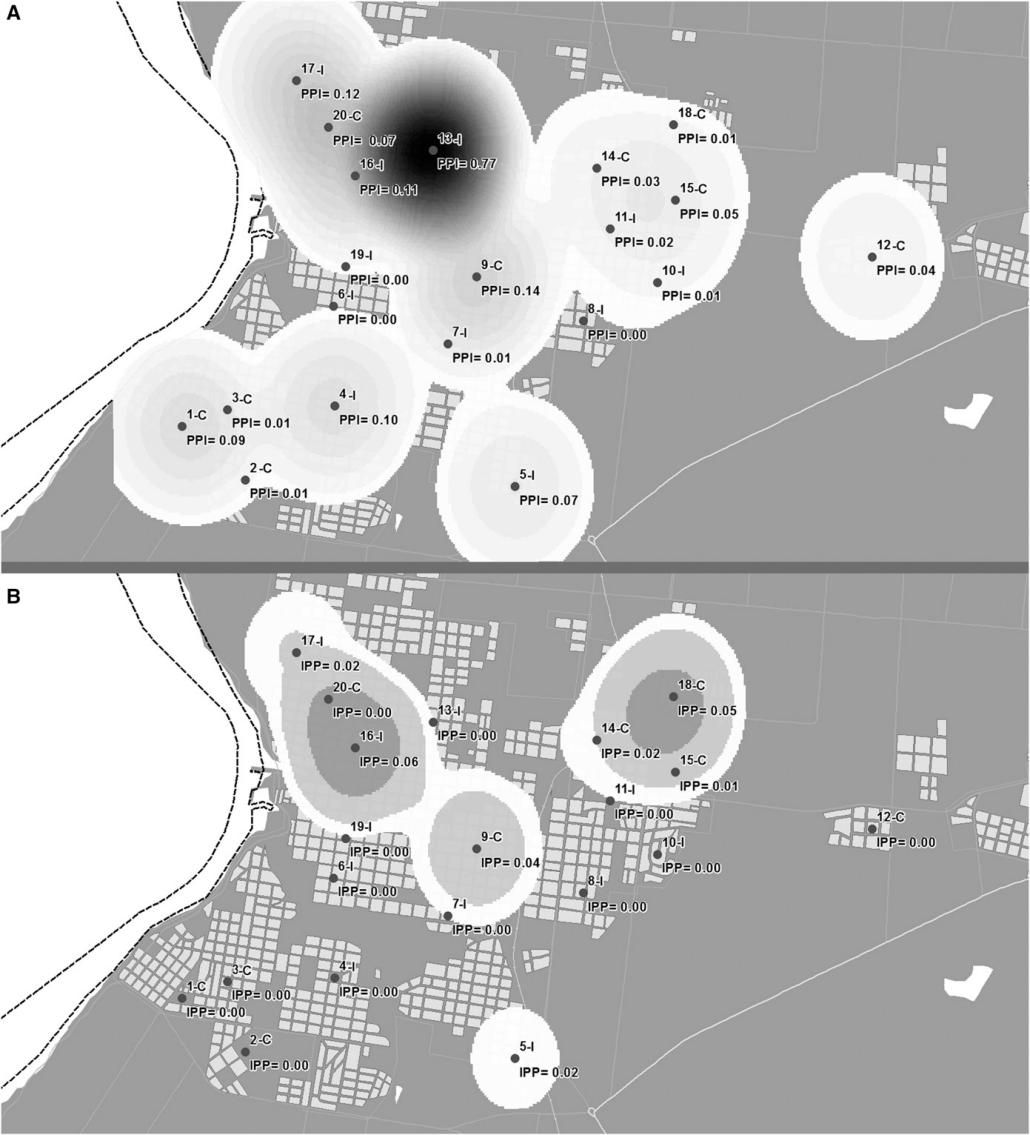
Pupae per person index representation in the city of Salto (Kernel method). (**A**) baseline (from April to May 2015); (**B**) follow up (November 2015). It was used data interpolation and data smoothing using the Gaussian kernel.^36^

The PPI values varied dramatically among the clusters, for both the baseline and the follow up surveys (Figure 2). Clusters 9-C (PPI = 0.14), 13-I (PPI = 0.77), 16-I (PPI = 0.11), and 17-I (PPI = 0.12) showed higher PPI values than others as they share common characteristics: abundant vegetation, houses with gardens, tree-lined streets, nearby parks and football stadiums and nearby small rivers. Although only 28% of the containers with unused water were reported in these three clusters, these containers produced 61% of all pupae collected.

### Process analysis and satisfaction surveys.

The household survey after the intervention covered 920 households in intervention clusters and 560 households in control clusters.

The overall acceptance of the intervention was 69% (631 households). The acceptance was particularly high in the area with community participation compared with the areas without community participation, overall acceptance = 78% versus 66%, respectively, *P* < 0.01; proportion of full bags handed back as percentage of the total of bags delivered = 59% versus 48%, respectively, *P* < 0.01. Radio was the most important source of information (72% of all information sources). In the area with community participation 18% the information was obtained from primary schools. This source of information was not mentioned in the other intervention clusters. Family and neighbors were mentioned as other information channels in all the intervention clusters.

Willingness to participate was extremely high (99% in intervention clusters and 97% in control clusters), because it was considered a good way to prevent dengue fever (98% of responses). In control clusters only 4% of the respondents had heard about the actions going on somewhere else in the city.

### Cost analysis.

The costs of the intervention activities in the scaling up process (without community participation) were 45.6% lower compared with the estimated costs of the routine activities executed by the Ministry of Health and the Municipality of Salto (cost per house attended USD 2.91 and USD 5.35, respectively). Lower costs were explained mainly by the personnel required. We estimated that the routine operations require 4,892 hours of work to cover an equivalent to 5,319 houses done by two vector control officers who enter the premises and collect and remove containers. Conversely, in the intervention approach the same number of control officers used 1,620 hours to visit the same number of houses to distribute trash bags for the community to remove the containers by themselves. Overall, the reduction in personnel costs was −53.86% (Table 3).

**Table 3:** Cost (US$) per house of implementing the scaling up process (with and without community participation), research project, and routine for vector prevention

	Scaling up process with community participation	Scaling up experience without community participation	Research project*	Routine
Capital				
Vehicles and equipment	0.02 (0.4%)	0.02 (0.5%)	0.00 (0%)	0.02 (0.4%)
Recurrent				
Personnel	2.09 (53.6%)	2.09 (71.9%)	1.89 (42.1%)	4.53 (84.6%)
Consumables: Information	0.24 (6.1%)	0.24 (8.2%)	0.88 (19.7%)	0.24 (4.5%)
Consumables: Source Reduction†	0.35 (8.8%)	0.35 (11.9%)	0.51 (11.3%)	0.37 (6.8%)
Consumables: Others	0.14 (3.5%)	0.14 (4.7%)		0.14 (2.6%)
Meetings	1.02 (26.1%)	0.02 (0.8%)	0.85 (19%)	0.01 (0.2%)
Transport	0.05 (1.3%)	0.05 (1.8%)	0.12 (2.7%)	0.05 (1%)
Training	0.01 (0.2%)	0.01 (0.2%)	0.23 (5.1%)	0.00 (0.1%)
Total	3.91	2.91	4.48	5.35

Value and percentage of participation of each component of each cost.

* Costs of the intervention package in a smaller scale research project.^33^

† Consumables are break down in information materials such as flyers and leaflets, source reduction materials such as the plastic bags distributed to households to collect water containers and other minor materials used for the intervention (e.g., folders).

In the intervention with community participation, personnel cost reduction was partly counter-balanced by other costs, mainly costs of community meetings. Routine activities costed on average USD 5.35 per house served but intervention activities with community participation were 26.9% lower (USD 3.91).

## DISCUSSION

Most dengue control efforts are based on the suppression of *Ae. aegypti* and not on vector elimination^43,44^ which is particularly important in countries like Uruguay where the vector has arrived but not yet the disease (with the exception of some recently reported cases). Targeting the most productive container types for adult *Aedes* mosquitoes will contribute to optimize labor efficiency, cost reduction, and maximum elimination of adults.^45,46^ Confirming the results obtained in a previous study in Salto,^34^ the current study was able to identify the epidemiologically relevant container types and their use. These were the discarded small water containers, low in numbers (10% of all containers), but producing 78% of pupae (as a proxy for adult mosquitoes).

The highest PPI values (PPI = 0.77), indicating the ratio between vectors and people, were obtained in the autumn survey during the season with the most elevated vector densities in Uruguay at a measured average temperature of 18°C. This would not be sufficient for possible DENV outbreaks according to the computer models by Focks and others^46^ (PPI = 7.13 at a temperature of 22°C), considering that DENV herd immunity in the population of Salto can be considered to be close to zero. It should be taken into account, however, that the Focks and others models^46^ establishing PPI thresholds for epidemic transmission have yet to be validated and that climate change can elevate ambiental temperatures favoring DENV transmission. Therefore, vector control as a preventive measure is important.

Even though scaling up novel vector control interventions is a complex process, 91% of the goal to reach 10,000 homes (one third of the city of Salto) was met. Community engagement and inter-sectorial partnerships for the prevention and vector control was achieved, which was the key to the success. Hindering factors were the following:

- Electoral processes at national and local levels during the scaling up activities created uncertainty. Even though the elected authorities ratified support, the financial and logistic difficulties at municipal level continued to affect the intervention activities.
- Only 58% of the homes visited could be contacted, due to absence of residents during day time. It will be necessary to adjust the timing of contacting people in their homes which unfortunately has cost implications.

Favoring factors were the following:

- A high percentage of the delivered bags (86%) were collected, indicating that when contact with the homeowners is made, the process is successful.
- Of households, 58% had un-used water containers in their homes despite the cleaning campaigns carried out in the city for several years and most of the remaining households had careful checked for those containers.
- Breeding places for *Ae. aegypti* were removed in about 50% of households visited. Adding the many additional bags with discarded containers supplied by neighbors, the removal of the majority of breeding places was achieved explaining the reduction of vector abundance (reduction of PPI) in the intervention clusters.
- The intervention was cheaper (−45.6%) than the routine activity applied by the vector control services.

When doing a large scale intervention, there is room for cost saving. For example, training and transport costs: training is mostly a fixed cost (largely independent of the number of trainees), while transport costs increase with the scale of the intervention, but not proportionally. Conversely, the major cost driver (personnel) increases almost proportionally with the scale of the intervention. It is worth noting, that there are also opportunities for reducing the costs of routine operations, for example, cost reduction for items such as “consumables, meetings, and personnel” simply by better planning.

Community mobilization and partnership approach increases the effectiveness in removing containers; this has been also demonstrated by other authors.^47,48^ To obtain the support of public-health authorities, and taking into account the cost increase caused by promotional activities for community participation (25.6%), it is important to underline the positive impact of this participation on the effectiveness of removing containers and on the acceptability of these activities. As a higher goal, community participation can contribute to empowerment if these processes take place over longer periods of time and are accompanied by the creation of opportunities and environments where issues of power and control are explicitly addressed.^49^

In the new scenario in Uruguay with the appearance of cases of autochthonous dengue in Montevideo (located 500 km south of Salto) in February 2016, a close cooperative relationship among project team members and national health authorities has been achieved which underlines the importance inter-institutional cooperation. The aim of this cooperation is to develop jointly action plans taking into account environmental space, bio-ecological, anthropological, logistic, and communication aspects. If this process is successful, the research results will inform public policies to address these issues in the whole Nation. Uruguay can also be considered a “case study” of temperate climates where *Ae. aegypti* is entering; and where the threat of dengue, Zika, and chikungunya is real in a scenario of climate change.
